# Knowledge Production on Congenital Chagas Disease across Time, Borders and Disciplines: A Comprehensive Scoping Review

**DOI:** 10.3390/tropicalmed8090422

**Published:** 2023-08-22

**Authors:** Elise Rapp, Marina Gold

**Affiliations:** 1 HESAV School of Health Sciences, HES-SO, University of Applied Sciences and Arts Western Switzerland, CH-2800 Delémont, Switzerland; elise.rapp@hesav.ch; 2 Institute of Humanities in Medecine, Faculty of Biology and Medecine, University of Lausanne (UNIL), CH-1015 Lausanne, Switzerland; 3 Fundación Mundo Sano, Buenos Aires C1061ABC, Argentina; 4 Institut für Sozialanthropologie und Empirische Kulturwissenschaft (ISEK), University of Zürich, CH-8005 Zurich, Switzerland

**Keywords:** congenital Chagas disease, mother-to-child transmission, vertical transmission, pregnancy, social science

## Abstract

Congenital transmission is a key route of *Trypanosoma cruzi* infection in Latin America and globally, contributing significantly to the burden of Chagas disease. The interruption of transmission from mother to child has recently become a focus issue. However, the research landscape on congenital Chagas disease remains largely unexplored. The purpose of this scoping review is to assess the production of knowledge on congenital Chagas disease (CCD), aiming to identify research trends and potential gaps. Our initial hypothesis was that the CCD literature overly represents the medical sciences and that there is a need for socio-cultural research on the subject. We conducted a systematic search of publications focusing on congenital Chagas disease in six languages (English, Spanish, Portuguese, French, German and Italian). This comprehensive literature search identified 876 studies that met the inclusion criteria, out of a total of 8893 sources. The relevant literature was analyzed by language, year of publication, discipline, source type and research location. The main outcome of this study has been to prove our hypothesis that there is a scarcity of knowledge produced within the non-biomedical sciences on CCD. This underscores the need for further exploration into the social and structural issues surrounding this disease. Visually clear data concerning congenital Chagas disease produced by this study can contribute to hone in future research efforts and support funding applications. Additionally, this article provides a reference list that other researchers can consult for their own studies.

## 1. Introduction

Chagas Disease (CD) is a neglected tropical disease transmitted by the parasite *Trypanosoma cruzi* that affects approximately 6 to 7 million people worldwide [1]. The vector is endemic in parts of south and Central America [2]. Argentina, Brazil, Bolivia, Paraguay, Colombia, Peru, Venezuela, Ecuador, Guyana, Central America, Mexico and the south of the United States are some (but not all) countries where CD is endemic. The transmission of *T. cruzi* primarily occurs as humans or other mammals come into contact with the feces or urine of infected blood-sucking triatomine insects. Other forms of transmission include blood transfusion from infected donors, the consumption of food or drinks contaminated with T. cruzi, organ transplants and congenital transmission during pregnancy or birth [3]. “In vector-free areas within and outside Latin America, mother-to-child transmission is currently the main mode of T. cruzi transmission” [4].

According to the World Health Organization [3], it is estimated that there are approximately 1,125,000 women of reproductive age in Latin America who are infected with *T. cruzi*. Among these cases, there is an incidence of 8668 congenital infections per year [4]. Concerning Chagas disease in general, estimates point to 68,000–123,000 possible infected people in Europe [5] and 200,000–300,000 in the USA [6]. This implies that CD has become a public health problem in places with a high incidence of migrants from endemic areas, such as Australia [7], Japan, North America [2] Western Europe [5] or urban areas in Latin America without vector presence. Cases of congenital transmission have been reported in Spain [8], the USA [9], Italy [10], Switzerland [11], France [12], Sweden [13] and Ireland [14]. In 2011, the number of congenital transmissions estimated to occur each year was between 60 and 315 in the United States and between 20 and 148 cases per year in Europe [4], where health policies designed to control the transmission of Chagas disease during pregnancy are generally severely underdeveloped [15]. 

Congenital infection occurs, on average, in 5 percent of children born from chronically infected mothers [16]. The majority of infected newborns are asymptomatic [17]. If the treatment of congenital newborns is delayed or postponed, “the disease can progress to the undetermined chronic phase, with a lower rate of treatment success” [18]. Therefore, the active screening of pregnant women who live, have lived or whose mothers have lived in Latin America is crucial to identify infected women and monitor their children at birth for infection, as well as all siblings. If a congenital infection is confirmed, both benznidazole and nifurtimox can be used to treat the newborn. The treatment is highly effective to cure newborns, with a cure rate of more than 95% [17], and presents fewer adverse effects compared to adult treatment. For more detailed information, please refer to [4,17]

Therefore, the diagnosis of infection in pregnant women and their newborns has become the standard of care, integrated into the Pan American Health Organization guidelines for the elimination of mother-to-child transmitted diseases (EMTCT Plus or ETMI-Plus for the Spanish acronym) for Syphilis, Hepatitis B and HIV.

Antiparasitic treatment is contraindicated during pregnancy due to the unknown risks it may pose to the fetus. However, new data have shown that the diagnosis and treatment of women of childbearing age before pregnancy provide not only benefits for the woman but also play a crucial role in preventing future congenital transmission [19,20]. Therefore, the WHO has shifted its approaches from focusing not only on the early detection and treatment of infected newborns but also on the active screening of girls and women of childbearing age to prevent congenital transmission [3]. More globally, the World Health Organization roadmap for neglected tropical diseases (2021–2030) includes the aim to eliminate congenital transmission of Chagas disease as a public health problem by 2030 [21].

Given this paradigm shift, research on congenital Chagas disease (CCD) is fundamental in supporting the goals laid out by international health institutions and provides insights on how to develop adapted healthcare implementation. Medical practice and knowledge production are closely linked, and it is now widely recognized that effective health strategies should rely on global and comprehensive approaches that encompass not only biomedical sciences but also diverse disciplines and key actors. Therefore, to elaborate effective and sustainable health interventions, the research landscape should incorporate various dimensions, including social, economic, cultural and environmental factors, and rely on collaborative efforts with diverse stakeholders, including concerned communities, civil society organizations, policymakers and healthcare providers.

An increase in focus on CCD demands a more in-depth understanding of the research landscape, language predominance, global distribution of research disciplines, source types and research locations. This is a fundamental contribution to knowledge intended to improve maternal and child health, by identifying gaps and foci of the current approaches. This enables the identification of the predominant centers of research in order to encourage collaborations and serves as an advocacy statement in the creation of awareness about this form of transmission and the importance of early detection as an effective public health campaign. It is not the purpose of this article to analyze the content of the literature reviewed, which will be the focus of forthcoming publications. We argue, however, that understanding the geographical, language and disciplinary context of the literature on CCD is a valuable contribution to the knowledge landscape on Chagas disease in general. By highlighting approaches that require reinforcement for designing effective and tailored health interventions, this research supports the consideration that effective and appropriate health strategies must address the root causes of health disparities and promote health equity, particularly for neglected diseases that have long faced deficiencies in research and public health implementation. 

Scientific knowledge production on Chagas has been the focus of several studies [22,23,24,25,26,27,28]. They point to an ever-increasing publication activity on the topic, which is, however, not exempt from controversy. Some of the issues noted include discrepancies between the need for public health strategies and a research focus stemming mainly from a biomolecular perspective [28]. Another noteworthy characteristic of the current literature is the biomedical predominance, with scarce social science contributions; some exceptions include [29,30,31,32,33,34], even when CD should be considered from a multi-dimensional perspective, contemplating social, economic, cultural and political factors [35]. 

There are other reviews with a more specific focus, such as Chagas cardiomyopathy [36]; health education approaches [35]; and clinical studies on treatments [1]. Among them, one study surveys knowledge production and research activity specifically on congenital Chagas disease (CCD), highlighting a significant lack of research production on this topic [37]. However, the authors focused on the epidemiological and diagnostic aspects of CCD and covered literature on humans during the period 2006 to 2017, limiting the search to sources written in English. They excluded several types of studies, such as case reports, review studies, dissertations, studies without epidemiological and diagnostic aspects and studies conducted in animals or in vitro.

The contribution of our review is a much broader scope in terms of the type of literature, time frame, study focus and language coverage (English, Spanish, Portuguese, French, Italian and German). Within the large—and growing—corpus of the Chagas-related literature, there has been notably little attention paid to congenital Chagas. The purpose of this article is to review the academic literature on CCD in order to highlight the foci and gaps within CCD research. Our initial hypothesis was that the literature on CCD was notably scarce but increasingly necessary in order to support the progressive attention given to CCD within public health and global health initiatives. Furthermore, within the limited corpus on CCD, we also hypothesized that social, cultural and political issues surrounding CCD are largely overlooked within most academic approaches.

The aim of a scoping review is to map the literature on a specific topic or research area and to establish key concepts, gaps, types and sources of evidence that inform practice, policymaking and research [38]. As comprehensiveness is at the core of scoping reviews [38], we considered this method to be the most appropriate to explore the existing published literature and map the research landscape on this topic. According to Arksey H. and O’Malley L. [39], a scoping review can serve various purposes, such as examining the scope, scale and nature of research activities in a given field; determining the relevance of conducting a systematic review; summarizing and disseminating research results; and identifying research gaps. 

This article presents partial results from an extensive scoping review project conducted on the CCD literature, aiming at better understanding the current state of the art on CCD knowledge production. This paper assesses the current state of research on CCD, considering the disciplinary approaches, places where research is conducted, types of populations studied, languages and years of publication in order to identify gaps and areas for future research and knowledge dissemination. Additionally, part of the scoping review has been the creation of a comprehensive database of publications on CCD and making a reference list available for other researchers’ consultation.

## 2. Materials and Methods

This scoping review was developed using the methodological framework proposed by Arksey and O’Malley [39], the further recommendations provided by Daudt, van Mossel and Scott [38], and the (PRISMA-ScR) guideline [40]. It focuses on the progressive development of research efforts across disciplines, topics, research locations and study populations. No time limit was imposed on the review search. The last systematic search was conducted on 24 February 2022. 

### 2.1. Research Question

The main research questions guiding this scoping review have been the following:

1. How is the current literature on CCD distributed geographically, thematically and by discipline?
2. What are the gaps within the scientific literature on CCD?

These questions provided the framework for the choice of keywords that determined our research (detailed equations and databases used are referenced in the supplementary material Table S1: Databases and Equations used in Literature Search) and the tools used to code and analyze the results. There are other questions that can be asked of the data produced by the review, and some will also be considered here, while others will be the focus of future publications:

1. What is the geopolitical configuration of the research teams?
2. What is the geopolitical configuration of the research sites and which population/countries are most studied?
3. What changes can be observed through time in terms of the volume of research, themes and political geographical distribution?
4. In which journals is Chagas-related research being published?
5. Who are the most prolific authors?
6. How do research domains co-create knowledge (translational research and bedside-to-benchtop research)?
7. What problems emerge through the classification of countries and areas as endemic/non-endemic?
8. How is “congenital Chagas” defined?

### 2.2. Data Source and Search Strategy

The search strategy was developed in collaboration with an experienced professional librarian at the HESAV School of Health Sciences, HES-SO, University of Applied Sciences and Arts Western Switzerland. A systematic search was conducted on 24 February 2022 in the following 13 electronic databases: Biblioteca virtual en salud; Bibnet.org; Cinahl; EMBASE; IBSS; JSTOR; Medes; PubMed; PsycINFO; SciELO; Sociological Abstract; Teseo; and Web of Sciences Core Collection. MeSH terms and thesauri or subject headings were used in the databases PubMed, Cinhal and Embase. 

No date restrictions were applied, and the literature search included publications written in the 6 languages understood by at least one reviewer: English, Spanish, Portuguese, French, Italian and German. Additional papers were searched via other sources: fellow researchers working in the field, the pearl growing strategy, Google Scholar. 

### 2.3. Study Selection

All citations were exported to the reference manager software Mendeley (Version 1.19.8). After duplicates were removed using both Mendeley’s electronic tools for deduplicating and manual deduplication methods, all the remaining citations were reported in a spreadsheet in Microsoft Excel. The two authors independently examined titles and abstracts for potential inclusion. After applying exclusion criteria, a total of 1289 references remained. At this stage, the list of articles that we could not obtain online or through institutional funds available to the authors (University of Zurich and University of Applied Sciences and Arts Western Switzerland) were sent to colleagues, along with a request for support in obtaining full texts. Our corpus includes all articles (including those not fully available, i.e., only accessible through their title or title and abstract). The full texts (when available), or abstracts, were examined independently. An important point was to analyze whether the CCD subject was a main focus of the publication. For this, we compiled a list of articles that we identified as not focusing solely on CCD but including other topics (other congenital diseases or Chagas disease topics). We then independently screened this list to identify publications with a significant focus on CCD. We included publications in which CCD is at least an important part of the article or provides significant or new information on the topic. Any doubt about the inclusion of an article was discussed by both authors. As our aim is to explore the extent of available literature, we did not assess the methodological quality of the articles included, which is consistent with the guidelines on scoping review methods [38]. 

During the coding process, we realized that some duplicates were not previously identified because they had different titles in multiple languages. Particularly, non-accessible articles may lack sufficient information or abstracts, making it challenging to determine their potential duplication. Electronic methods of deduplicating search results have limited effectiveness [41], and, despite additional manual searches, duplicate articles with different titles but similar content may have gone unidentified. Given these challenges, at a late stage in the process, we decided to involve a librarian colleague to perform an external review and clean-up of the sources, specifically focusing on identifying duplicates. This step was deemed necessary due to the substantial number of sources analyzed and the difficulties encountered in obtaining many articles. 

### 2.4. Eligibility Criteria

Titles and abstracts were screened independently by the 2 authors using the following inclusion/exclusion criteria:

### 2.5. Data Coding

The two authors divided the articles according to language and coded them individually in an online shared Microsoft Excel spreadsheet. To ascertain congruence, a “standardized data extraction form” [42] was developed. Thirty randomly selected publications were extracted and independently reviewed by the two authors and compared, as a way to ensure the consistency of the coding criteria and adjust it, when necessary. Based on this review, and regular weekly meetings between the authors, the coding categories were defined and further uncertainty about the classification of a source was discussed by both authors until agreement was reached. In addition, during the coding process, twenty randomly selected publications were coded by both authors and compared to see if the categories were consistently coded. Among unavailable sources, the maximum possible data were collected through available abstracts and, in some cases, from the title, if it was informative enough.

The following data were extracted from the selected sources: the year of publication, language of publication, disciplines, place of research (for articles that are not knowledge syntheses), endemic/non-endemic categorization, studied population and types of literature. While the year and language were straightforward categories, we encountered some categorical issues in most other codes. Upon deliberation, and based on our research interest, we determined the following definitions:

Discipline: As our aim was above all to explore the knowledge produced and the disciplinary field for which the research is intended, we decided to code the disciplinary fields rather than to perform a bibliometric analysis of the authors’ location and the disciplines in which they are registered. Some sources were classified as involving more than one disciplinary approach. 

Place of research: This category encompasses publications with primary data generation and excludes knowledge synthesis and commentaries. Place of research with original data production include laboratory research, clinical research, clinical cases, case studies (research outside of hospital), field research (veterinary research), mathematical modelization and data analysis (including a systematic review of the literature if they include a meta-analysis creating new knowledge). Where mentioned, we collected the research locations (the hospital or region where the data were collected, the location of the laboratory, the country from which the data are collected, field sites).

Endemic versus non-endemic: We encountered epistemic questions in attempting to distinguish research places according to the evolving spatial and temporal reality of endemicity. What terms should we use to identify research conducted in non-endemic areas but in so-called “endemic” countries? How should countries that are no longer endemic but located in the southern part of the American continent be referred to? (e.g., Chile since 1999). In an effort to highlight the complexities of the distinction between endemic and non-endemic, and observing the discrepancies between the terms used and the multiple socio-medical realities of different Latin American countries, we proceeded in the following manner:

1. We identified the country and city where the research was conducted in a column called “research location”;
2. We identified whether the country is endemic or non-endemic;
3. We then subclassified endemic countries into whether the area of research is endemic or not (NEA—non-endemic area);
4. We considered whether the endemicity status has changed through time.

The comparison of the latter two columns allows us to analyze the discrepancy between the terms used and the multiple socio-medical realities of different Latin American countries. A more thorough analysis of this issue will be the focus of a future paper. 

Studied Population: This refers to the subjects of study. The larger distinction was between animals and humans, as much of the research was laboratory trials on rats and mice. But within the human cohort there were subclassifications when the research was conducted in the laboratory on body organs, cell tissue, blood samples and other body products separate from identifiable clinical patients.

The characteristics of the sources were graphically mapped using Microsoft Excel. Depending on the needs of the analysis, the unavailable articles are included or not. For each result and graph, we indicate the data included in our analysis (fully available or limited corpus).

## 3. Results

The systematic literature search yielded 8893 sources from databases and 22 sources from other methods (Google Scholar and the pearl growing strategy). The corpus was reduced to *n* = 3409 sources after removing 5484 duplicates. After examining the title of the remaining sources, 1462 studies were excluded, leaving 1947 sources that were selected based on their abstracts for further reading. Of these, 1302 sources (1289 from database searches and 13 from hand searches) were sought for full-text retrieval. All 1302 sources (full text or only with the abstract or title available) were further examined according to the inclusion criteria of CCD being at least an important part of the publication or that the publication provides significant or new information on the topic. At this stage, an external review identified 23 duplicates that had not been previously identified because their titles were in different languages. A total of 876 relevant publications fit the categorizations above (Table 1). Among them, 211 sources were not accessible online through the institutional funds available to the authors or through fellow researchers. The majority of unavailable sources (*n* = 151; 71.5%) were published before the year 2000. Of these inaccessible sources, 126 had available abstracts, which enabled the collection of information. A total of 85 sources were not available online and had no abstract. In some cases, the title was informative enough to extract some basic data. 

**Figure 1:**
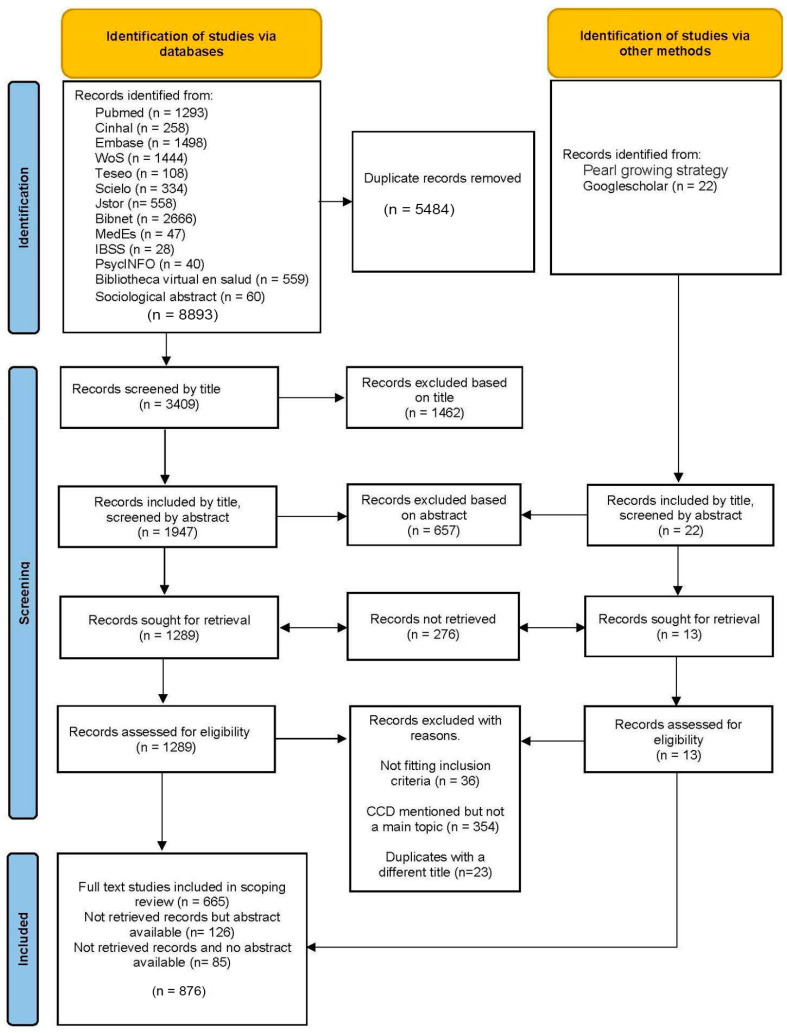
PRISMA diagram of study selection process adapted from [40].

The process of article selection is presented in the PRISMA flow diagram (Figure 1).

### 3.1. Publications by Language

**Figure 2:**
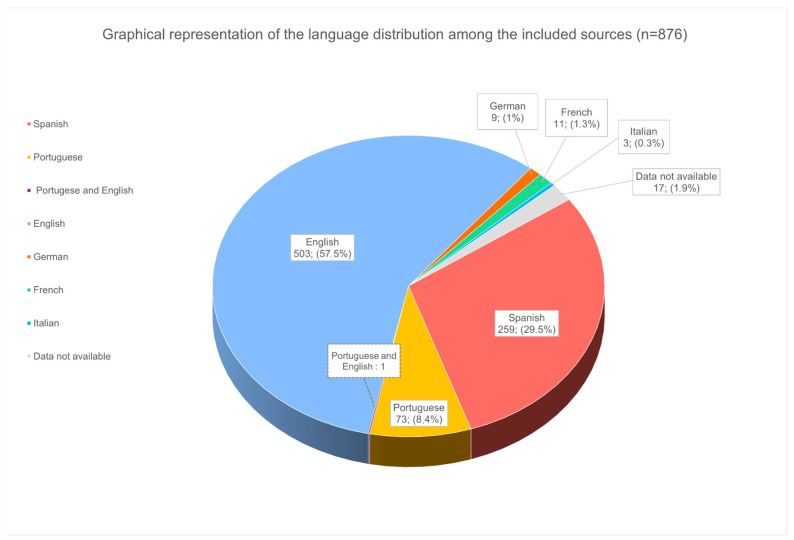
Graphical representation of language distribution among the included sources. This graph takes into account the entirety of the data set (*n* = 876). The percentages representing the language distribution within the entire corpus are calculated based on a division by 877, taking into account that one article is written in 2 languages.

The languages of the publications, in order of prominence, are as follows: English = 504, Spanish = 259, Portuguese = 74, French = 11, German = 9 and Italian = 3 (Figure 2). Of these articles, one is written in Portuguese and English within the same article and eight (2 × 4) sources are identical articles published in two different languages: two in Portuguese and English and two in Spanish and English. A total of 17 (1.9%) sources (amongst those for which we had no access to the full text) had no data available on language. There is a predominance of English publications (*n* = 504, 57.5%), even while Chagas affects mostly Spanish-speaking countries. 

### 3.2. Publication by Year

**Figure 3:**
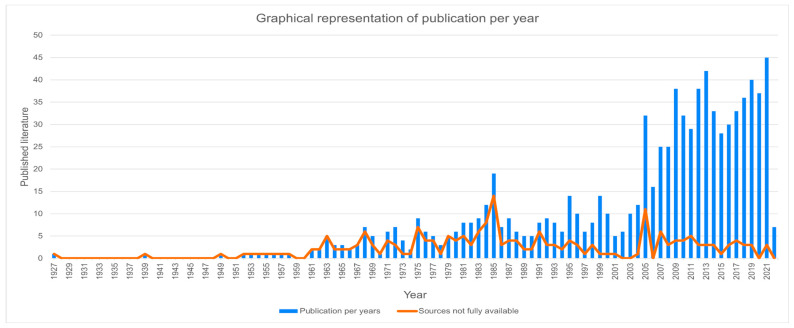
Graphical representation of publication per year (up to 24 of February 2022) and article availability (September 2022) for authors (ER and MG) according to their institutional access (University of Zurich and University of Applied Sciences and Arts Western Switzerland) and through their professional networks. This graph takes into account the entirety of the data set (*n* = 876).

The first publication appears in 1927 (Figure 3), with a sporadic and slight increase in material from the 1960s onwards. The vast majority of the sources (64.6%; 566/876) were published since the year 2005. Most of the unavailable sources (71.5%; 151/211) were published before the year 2000.

### 3.3. Publication by Disciplines

**Figure 4:**
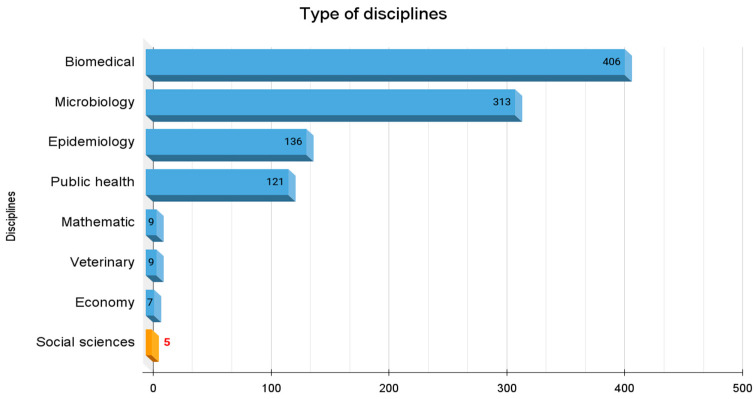
Quantification of the type of disciplines in which the sources were classified. This graph takes into account the entirety of the data set (*n* = 876).

**Figure 5:**
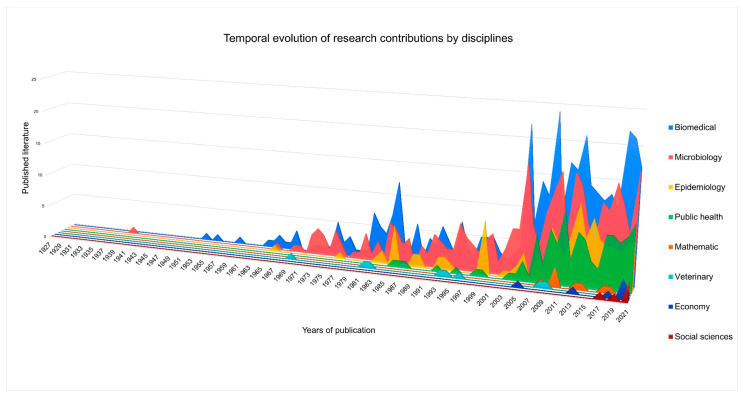
Temporal evolution of publication by disciplines. This graph takes into account the entirety of the data set (*n* = 876).

Upon analyzing the publications by discipline, it becomes evident that a significant proportion of approaches belong to the biomedical and microbiological fields, while a smaller proportion are from epidemiology and public health. Disciplines such as mathematics, economics, veterinary science and social sciences are comparatively underrepresented, as clearly seen in Figure 4. Moreover, these disciplines appear in more recent years, as depicted in Figure 5. The sum of the disciplines appears greater than the total number of publications in our corpus (*n* = 876) because some of the sources were classified as involving more than one disciplinary approach. 

The classification of the literature by research discipline has been outlined in more detail in our Excel database. In order to facilitate the representation of research by disciplinary field, different types of disciplines have been grouped together into broader sets. For example, the term “biomedical” includes disciplines such as gynecology, ophthalmology, clinical cases, pediatrics, etc.; “microbiology” includes parasitology and genetic research; “epidemiology” includes serological prevalence studies; and “public health” includes studies that often focuses on implementation and policy. “Mathematics” includes different modeling techniques based on available data, such as modeling the transition from vector transmission to vertical transmission in Chile [43] or the detection of cluster studies in the occurrence of Chagas disease in puerperal women in the state of Minas Gerais, in Brazil’s southeast [44]. “Veterinary” studies focus on T. cruzi analysis in wild animals, such as bats [45], monkeys [46,47,48] and birds [49]. The “economic” studies include cost-benefit analyses of screening programs [50,51]. The five social science [52,53,54,55,56] papers fall within anthropology, sociology and interdisciplinary teams that include anthropologists. To be clear, there are significantly more social science articles on Chagas, but only these five specifically focus on the topic of congenital Chagas or have a significant portion of the article dedicated to congenital Chagas according to our inclusion criteria.

### 3.4. Publication by Source Type

**Figure 6:**
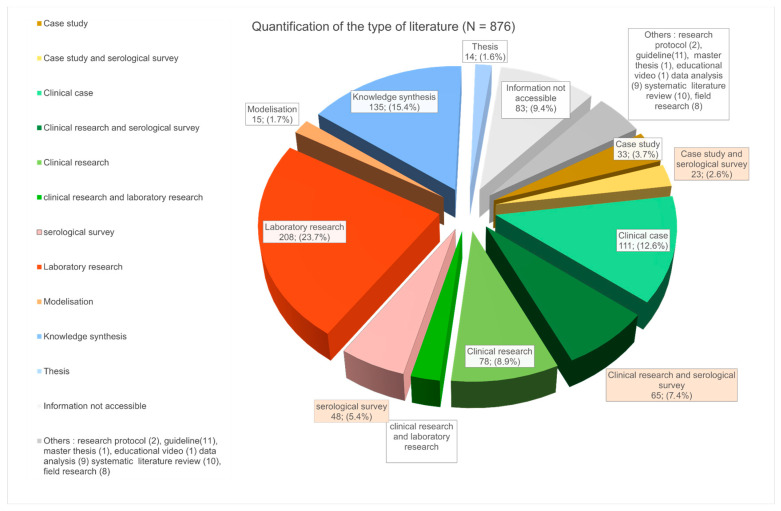
Types of publications and distribution. This graph takes into account the entirety of the data set (*n* = 876).

The distribution of literature types shows a predominant contribution from the healthcare sector accounting for 31.4% (*n* = 275) of the total. This includes research conducted in hospitals, which represents 18.7% (*n* = 164) of the literature, and clinical cases, which contribute 12.6% (*n* = 111). These figures are represented by different tones of green in Figure 6. Additionally, laboratory research publications constitute 23.7% (*n* = 208) of the overall distribution. It is noteworthy that serological surveys (15.5%; 136), which represent epidemiological data collection, constitute an important part of the publications from clinical fields as well as from outside hospitals (defined here as case studies). We can observe that the literature referred to as “general knowledge production”, including literature synthesis and commentary, represents a substantial proportion of total publications (15.4%; 135). Those publications aim primarily to synthesize and disseminate knowledge.

### 3.5. Publications by Research Locations

**Figure 7:**
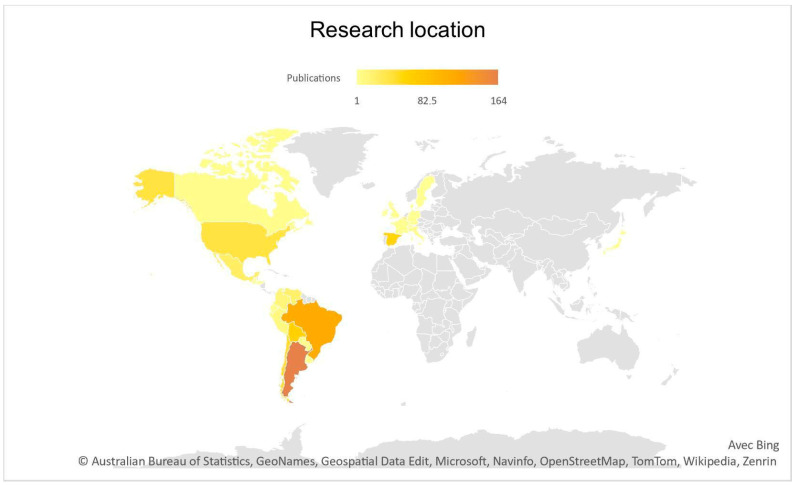
Mapped representation of the countries where the research was performed. This graph takes into account the data set among primary research publications based on original data (*n* = 641): laboratory research, clinical research, clinical cases, case studies (research outside of hospital), field research (veterinary research), mathematical modelization, data analysis and original protocols. Same publications written in 2 different languages were counted for 1 source.

An analysis of the 641 primary research publications based on original data reveals that research on congenital Chagas has been conducted in 27 countries. Argentina (164; 24.3%) and Brazil (112; 16.6%) are the two most represented countries, followed by Bolivia (63; 9.35%), Spain (59; 8.75%) and Chile (50; 7.4%) (Table 2). The map (Figure 7) does not represent the distinctions between endemic and non-endemic areas within countries. 

This Table takes into account the data set among primary research publications based on original data (*n* = 641): laboratory research, clinical research, clinical cases, case studies (research outside of hospital), field research (veterinary research), mathematical modelization, data analysis and original protocols. The same publications written in two different languages were counted for one source. The sum of the countries (*n* = 674) appears greater than the total number of primary research studies (*n* = 641) because some of the research was conducted in more than one country.

### 3.6. Publications by Subject of Study (Animal/Human/Body Part)

**Figure 8:**
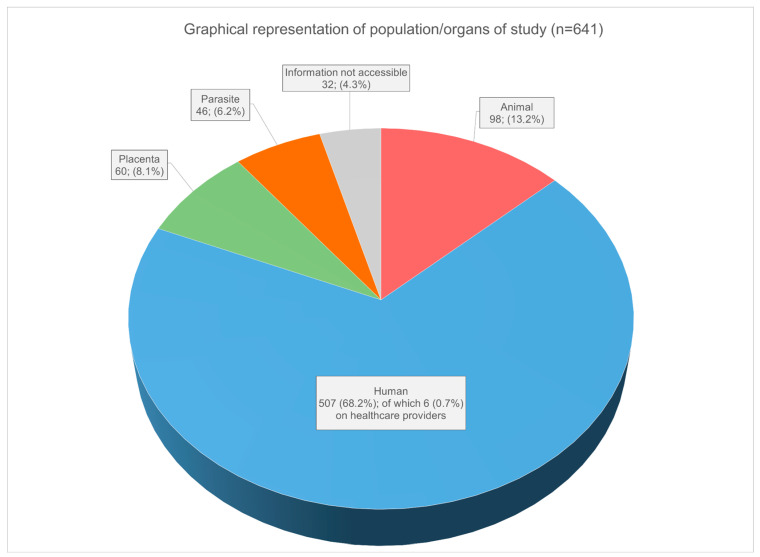
Graphical representation of subjects of studies (populations and organs) among research studies. This graph takes into account the data set among primary research publications (*n* = 642) based on original data: laboratory research, clinical research, clinical cases, case studies (research outside of hospital), field research (veterinary research), mathematical modelization, data analysis and original protocols. Same publications written in 2 different languages were counted for 1 source. The sum of the subjects of studies (*n* = 674) appears greater than the total number of primary research (*n* = 641) because some of the research was classified with more than one subject.

The vast majority of studies (507; 68.2%) focus on humans, while (98; 13.2%) examine animals, (60; 8.1%) of studies specifically target the placenta, and (46; 6.2%) are dedicated to the T. cruzi parasite. These last three categories are predominantly studied in laboratories. Among research focused on humans, the majority comprises pregnant women and/or children and a very small percentage of studies (*n* = 6 0.7%) specifically involve healthcare professionals. A significant portion of the research (60; 8.1%) focuses on the placenta (both human and animal) rather than the human/animal as a whole. The vast majority of them focus on the immune system of the placenta in order to better understand the mechanisms of parasite invasion. To illustrate the significant effort research devoted to the placenta, in Figure 8 we have distinguished studies focusing on placenta from those encompassing whole humans and animals. 

## 4. Discussion

This research is the first systematic attempt to assess the global scope of CCD across six languages. In light of the increasing production of the literature on Chagas disease (CCD), this paper explores the knowledge production landscape on CCD. Through an analysis of geographical, language, field of research and disciplinary aspects, it examines the scope of the literature dedicated to CCD. More specifically, this study examines the hypothesis that the literature on CCD is mainly produced within the biomedical sciences, highlighting the need of adopting a global perspective in health strategies and recognizing the complex interaction of social, economic, cultural and environmental factors. This research/publication is part of a broader project exploring the current worldwide state of the art on CCD knowledge production. 

Figure 3 clearly demonstrates that the interest in CCD has increased in the last two decades of the twentieth century. In the mid-1980s, there was a peak in CCD publications emerging from Brazil, Chile and Argentina, but these slowed down in the 1990s. This increase can be explained by the Tropical Disease Research (TDR) grants provided by the WHO in the 1980s for training and research on neglected tropical diseases. TDR, the Special Programme for Research and Training in Tropical Diseases, is a global program of scientific collaboration that helps facilitate, support and influence efforts to combat diseases of poverty. It is co-sponsored by the United Nations Children’s Fund (UNICEF), the United Nations Development Programme (UNDP), the World Bank and the World Health Organization (WHO). They awarded PhD scholarships and research grants to various researchers in Argentina, Brazil and Chile, increasing the focus and output of research on Chagas. This initiative catalyzed the research in this domain. Publications increased again considerably since 2005, as did the variability in the types of publications (Figure 6). This increase is likely to be sustained in the coming years, given the fieldwork and policy transformations foreshadowed by the Iberoamerican Initiative for the elimination of congenital Chagas organized by the Secretaría General Iberoamericana (SEGIB) and endorsed by the Pan American Health Organization [57]. The increase in publications since 2005 in the areas of biomedicine and microbiology can also be related to the improvement of molecular techniques and rapid tests for the diagnosis of infection both in the mothers (during pregnancy or upon admission to hospital for childbirth), newborns (for follow-up of treatment in neonates) and infants.

A negative correlation between the availability of articles and the amount of time that has passed since their publication can be observed (Figure 3), suggesting that older articles tend to be less accessible. This indicates that, in the present day, not only is there a larger quantity of articles being published but also a greater percentage of them are available online. Conversely, there is a stagnation in the number of unavailable articles based on their year of publication. This analysis reflects all sources (including those not available online). As for the variability of the data, it reveals a consistency between a markedly biomedical concern with Chagas diseases as the bulk of the literature after 2000 has been laboratory research, clinical case reports, case studies or knowledge synthesis papers. Secondary data production, referred to as “general knowledge production”, including literature synthesis and commentary, represents a substantial proportion of total publications. This intention to disseminate knowledge beyond individual research efforts underscores a commitment to fostering knowledge circulation and raising awareness of CCD. 

Interestingly, while the healthcare sector is an important contributor to literature production, our review reveals that a very restricted proportion of studies focuses on healthcare providers themselves. Yet, these studies would be particularly important for identifying aspects (both logistical and emotional) that contribute to the difficulties encountered in clinical practice. This is particularly relevant in non-endemic areas, where barriers to access and care often fall within the lack of knowledge and experience of health professionals with Chagas disease generally. Despite the growing interest in CCD, there are still many gaps in knowledge that need to be filled, particularly in the domain of social science. Indeed, saliently, the review shows that the literature stemming from the social sciences is very limited. Yet, Chagas disease (and its consequences) extends far beyond the medical sphere, encompassing social, economic, cultural and political dimensions that need to be more thoroughly understood and documented. In fact, there are a growing number of publications focusing on the social and cultural elements of Chagas disease, as mentioned in the introduction. Furthermore, within interdisciplinary teams, social science, as well as other less prominent sciences, such as veterinary sciences, are often represented, yet mostly within projects framed by a biomedical perspective. Additionally, these do not focus specifically on the issues pertaining to the congenital aspect of CD. Such research is clinically oriented and focuses on how to increase access to care. The perspective of affected people is often absent and the multiple social determinants of health remain unexplored. A comprehensive approach to tackling Chagas disease requires considering the perspectives and experiences of affected individuals and exploring Chagas disease more broadly as a social phenomenon embedded in complex contexts. For better understanding the dynamics surrounding CD, social sciences are crucial to develop interventions tailored to the specific needs and realities of the people and communities affected. In this sense, social science approaches have much to offer. 

Other key actors notably absent from the corpus are patient associations. Yet, to date, thirty associations of people affected by CD have been reported on five continents [58]. Patient associations play a crucial role in raising awareness about CD, reducing stigma and promoting community engagement. Their insights and expertise should be taken into consideration when shaping educational programs, communication strategies and public health initiatives to enhance their effectiveness, cultural sensitivity and responsiveness to the specific challenges faced by people affected by Chagas disease. Collaboration and information sharing among researchers, healthcare providers and patient associations should lead to more comprehensive and effective approaches to tackle Chagas disease. Therefore, more publications focusing on the involvement of patient associations providing insights into their experiences and best practices, and the impact of their activities could contribute greatly to the knowledge landscape on Chagas disease. 

Some authors raise concerns about the practical use of knowledge gained from the growing number of international publications stemming mainly from a biomolecular perspective [24]. Questioning if such research is translated into effective uses to solve socio-medical problems [28], they describe the predominance of microbiological and biomedical approaches as belonging more to a trend within epistemic communities than to a specific concern for solving health problems in affected countries. Other authors point out that the social sciences should play an important role in understanding the complex socio-cultural, economic and political issues associated with diseases [33] but that knowledge production on neglected diseases is often neglected by these disciplines [59,60]. However, the purpose of this article is not to determine which of the biomedical sciences and social sciences is responsible for the epistemological imbalance observed in this scoping review but rather to call for an integrated approach that incorporates the perspectives of both biomedical and social sciences to address complex issues related to CCD more effectively.

Chagas should be considered from four interrelated dimensions [35]: biomedical, epidemiological, socio-cultural and political. Chagas disease is often associated with stigma and discrimination, and people at risk do not always want to be diagnosed and/or receive treatment. These aspects are likely to be complicated in the context of pregnancy, a significant life transition that can introduce various psychological stressors for women. Moreover, pregnancy is an entry point to the medical system, providing a privileged opportunity for diagnosing this disease that is often silent for decades. A diagnosis of Chagas disease is an additional stressor that can further exacerbate existing sources of stress (physical changes, financial pressures, relationship adjustments, concerns about the health and well-being of the baby and the anticipation of becoming a parent). Further, pregnant women do not have direct control over the infection process during pregnancy. The uncertainty and potential outcomes of the infection on their baby can lead to heightened levels of anxiety and distress. It is important to understand the experience of screening to support the emotional and psychological well-being of women during pregnancy. 

The gap between the biomedical vision for patients and the actual lack of acceptability for screening and treatment call for more research on understanding the socio-cultural aspects surrounding the disease. Social sciences provide a systematic and rigorous approach to studying human behavior and society, gathering data on the experiences, attitudes and behaviors of different populations. This helps to identify patterns and trends that shape people’s lives. Furthermore, social sciences provide insights into the ways in which structural and cultural factors influence people’s experiences and perspectives, to understand complex issues from multiple perspectives and to develop nuanced understandings of the experiences of different populations.

Some issues related to Chagas disease are very specific to the context of pregnancy. For example, the risk factors that determine the transmission of the parasite to the fetus are not well-known. There is also no scientific certainty regarding the risk of disease transmission during breastfeeding, although the risk of transmission of the disease to breastfed babies is believed to be negligible. Maternal Chagas disease is generally not considered a contraindication to breastfeeding, except for women in the acute phase of the infection or those with bleeding nipples [17]. In this context of scientific uncertainty, it would be important to explore the psychosocial dimension of breastfeeding women as well as healthcare professionals and how they manage communication about this topic. The psychosocial burden that CCD means for pregnant women, as they manage feelings of guilt and the responsibility of passing on a disease to their children, has not yet been explored. Some work has been performed on the psychological burden of mothers with HepC [61] and HIV [62,63], but the socio-cultural conditions of Chagas require a slightly different analysis to understand what CCD means for those suffering it. Recent studies have demonstrated that the congenital transmission of Chagas disease can be prevented through screening and treating affected women of childbearing age prior to pregnancy [64]. However, considering that the treatment for Chagas disease can potentially have significant side effects, it would be important to document the perspectives and experiences of women undergoing this treatment. Those considerations highlight how conducting further research with a qualitative approach would be crucial to comprehensively document the perspectives of pregnant and breastfeeding women and women of childbearing age in relation to CCD. A comprehensive understanding can help in developing interventions that not only focus on the medical aspects but also take into account the emotional experiences of women living with Chagas disease. By considering the emotional well-being and experiences of women, interventions and policies can be tailored to provide appropriate support, counseling and resources to address their specific needs. Importantly, those approaches should not only provide valuable insights into the psychosocial dimension but also shed light on the broader contextual and structural aspects that influence the experience of the disease and its medical follow-up. 

Similarly, the scarcity of economic studies on the cost-effectiveness of treating pregnant women and their children is a gap that must be redressed [65] as it is crucial information in the recruitment of funds and political will to enact new policies on mother–child health. Furthermore, it is important to explore the socio-political dimension that explains the lack of CCD surveillance systems in non-endemic countries, where other routes of transmission are often controlled. These situations highlight how the health of migrants is influenced by various structural aspects related to the integration policies of migrants and the neglect of certain minorities, including undocumented migrants [66]. 

The research location predominance corresponds mostly to the endemicity of the countries (see Figure 7 and Table 2), with Argentina (24.2%) being the focus of the most amount of research, followed by Brazil (16.6%), Bolivia (9.3%) and Chile (7.4%). Brazil and Argentina are two countries described by Levin and co-authors (2021) as having a strong tradition of research on Chagas disease, with a predominance of molecular (immunology) approaches. Spain also represents a large percentage of research (8.7%), as a country receiving a high number of Latin American migrants, which has demanded of the local health system sensibilization towards the issue of Chagas. Furthermore, there are multiple programs in Spain (Barcelona, Madrid, Murcia) targeting Chagas generally and congenital Chagas specifically. A limitation of this data representation is that by exploring research location instead of bibliometric analysis of authors we are unable to show which country/place/research institutions or groups are more committed to Chagas research or likely to obtain funding for it. Instead, we portray simply the geographic location where the research is carried out and the data collected.

As Chagas disease is a neglected tropical disease, it is particularly important to understand how knowledge is produced. Firstly, it helps to identify areas where further research is needed. In this case, we have seen that research in areas such as the social sciences or economics sciences is scarce and that supplementary efforts are needed in those domains. Understanding how research is produced (which disciplines and types of literature are predominant and which are overlooked) is essential to addressing this neglected disease. The lack of knowledge about the systemic challenges faced by at-risk populations hinders effective action at the socio-political level. Secondly, understanding knowledge production can also help to identify areas where current knowledge is limited or contradictory, which may have important implications for clinical practice or public policy. This can inform future research projects and funding decisions and help prioritize research efforts and allocate resources more efficiently. 

Finally, by providing a comprehensive list of publications on CCD for future research purposes (Excel file S2), the aim of this scoping review is to contribute to the current research effort to make science more open. Open science is a global effort to increase the transparency and accessibility of scientific research, data and publications. This not only facilitates progress in scientific discovery but also promotes collaboration, replicability and accountability within the scientific community. This effort is also a contribution to reverting the neglected status of Chagas, by aiming to make research about it more accessible and raising awareness among the scientific community of the need to address congenital Chagas disease. 

We provided a comprehensive review of the published literature with the aim of being as thorough as possible. This exhaustive search required significant time from both authors, and the results do not include the analysis of article content, which is currently underway. 

Practical constraints were encountered, such as not accessible resources, limiting the depth and accuracy of our analysis. However, by considering the titles and abstracts of the unavailable articles, in many cases we could incorporate relevant information and insights into our analysis. Nonetheless, by combining the bibliographic access rights of our institution, we had access to a wide range of literature. Thus, this research provides a strong overview of the available knowledge that researchers and other stakeholders rely on to contemplate potential solutions. 

## 5. Conclusions

Through the inclusion of publications in six languages using peer-reviewed journals and other sources, this review provides a comprehensive overview of the literature landscape and the research on congenital Chagas disease in all major geographical regions of the world. In this article, we analyzed the extent, scope and nature of research activity related to CCD and discussed gaps in research approaches and disciplines. We have discussed the crucial need of further exploration in the fields of social science, specifically focusing on the experiences of pregnant and breastfeeding women and women of childbearing age living with Chagas disease, as well as the medical and systemic challenges they may encounter.

Two important themes have been left underexplored in this paper and will be the focus of future publications: the complexity of the issue of endemicity and the content and tendencies in the production of knowledge. A future publication will focus on the content produced by the literature, specifically on the socio-cultural aspects of CCD. 

Through the inclusion of publications in six languages using peer-reviewed journals and other sources, this review provides a comprehensive overview of the literature landscape and the research on congenital Chagas disease in all major geographical regions of the world. In this article, we analyzed the extent, scope and nature of research activity related to CCD and discussed gaps in research approaches and disciplines. It should be noted, however, that we did not assess the methodological quality of the articles included, which is consistent with the guidelines on scoping review methods [38] We have discussed the crucial need of further exploration in the fields of social science, specifically focusing on the experiences of pregnant, and breastfeeding women and women of childbearing age living with Chagas disease, as well as the medical and systemic challenges they may encounter.

In a forthcoming article, we will focus on the research results from the published literature, providing a summary of the knowledge produced and identifying major tendencies around the diagnosis, treatment and management of congenital Chagas disease.

## Figures and Tables

**Table 1:** Inclusion and exclusion criteria.

Inclusion Criteria	
Language	English, Spanish, French, Italian, Portuguese, German
Source Type	Peer-reviewed journals, theses, reports, books, policy papers, published commentaries and replies
Relevance	Be exclusively about congenital CD; otherwise, the section on congenital CD must be at least a significant part of the article or must provide significant or new information on the topic
Scope	Studies on pregnant women or women of childbearing age and studies that focus on children up to the age of 1 year (“time of general follow-up/monitoring, treatment”). Later-age studies may be included if the origin of the infection is identified as congenital. Studies with the title “congenital Chagas” even if the report concerns an adult.
Exclusion Criteria	Conference proceedings and paper abstracts, as we determined that we could not extract enough information from these and assumed that the worthwhile conference papers would have eventually resulted in publication.

**Table 2:** Representation of countries where research was performed.

Place of Research	Number of Publications	% of Production	Clinical Cases
Argentina	164	24.3%	20
Brazil	112	16.6%	23
Bolivia	63	9.35%	0
Spain	59	8.75%	16
Chile	50	7.4%	10
USA	39	5.8%	7
Belgium	25	3.7%	0
Mexico	24	3.6%	4
Venezuela	21	3.1%	1
Colombia	15	2.2%	3
Paraguay	11	1.6%	0
Peru	10	1.5%	1
Italy	6	0.9%	1
Canada	5	0.7%	3
Honduras	5	0.7%	1
Switzerland	5	0.7%	2
Ecuador	5	0.7%	0
El Salvador	4	0.6%	0
France	3	0.45%	1
UK	3	0.45%	1
Uruguay	3	0.45%	1
Germany	2	0.3%	0
Ireland	2	0.3%	2
Sweden	2	0.3%	2
Denmark	1	0.15%	0
Guatemala	1	0.15%	0
Japan	1	0.15%	1
Americas (Systematic Review)	1	0.15%	0
Worldwide Survey	3	0.45%	0
Data not available	32	4.75%	11

## Data Availability

In the supplementary material we are providing the list of references used to conduct this research, as well as their availability. All references were accessed online through the access granted by institutional affiliations to online resources.

## Supplementary Materials

The following supporting information can be downloaded at: https://www.mdpi.com/article/10.3390/tropicalmed8090422/s1. This publication is accompanied by Table S1: Databases and Equations used in Literature Search, and an Excel file (S2-Full Reference List) displaying the full list of references used for this review with the following data: author, publication date, title, language, journal and source availability.
