# Supplementary material for: Knowledge Production on Congenital Chagas Disease across Time, Borders and Disciplines: A Comprehensive Scoping Review
Source: Trop Med Infect Dis. 2023 Aug 22;8(9):422. doi: 10.3390/tropicalmed8090422 (PMC10536740; doi:10.3390/tropicalmed8090422)
Supplement: Supplementary file 1 [file tropicalmed-08-00422-s001.zip › S1 - Table S1_ Databases and Equations used in Literature Search.pdf]

Table S1. Databases and Equations used in Literature Search

Knowledge Production on Congenital Chagas Disease across Time, Borders and Disciplines: A Comprehensive Scoping Review

E.Rapp; M. Gold 2023

Data sources	Query
BIBLIOTECA VIRTUAL EN SALUD	Search by title : (cruzi OR chagas OR Trypanosom*) AND (prenatal OR ginecología OR embarazo OR embarazada OR madre OR congénito OR transplacentaria OR bebé OR bébés OR recién nacido OR neonat* OR feto OR pediátric* OR pré-natal OR ginecologia OR gravidez OR grávida OR mãe OR congénita OR transplacental OR criança OR crianças OR recém-nascido OR fetus OR gynecology OR pregnancy OR pregnant OR mother* OR congenital OR infant OR infants OR newborn* OR baby OR babies OR fetus OR pediatric* OR paediatric*)
BIBNET.ORG	(cruzi OR chagas OR Trypanosom*) AND (pränatal OR Gynäkologie OR Schwangerschaft OR schwanger OR Mutter OR kongenital OR transplazentar OR Säugling OR Neugeborene* OR Baby OR Kleinkinder OR Kinder OR Neonat* OR Fötus OR Pädiatrie OR prenatal OR gynecology OR pregnancy OR pregnant OR mother* OR congenital OR transplacental OR infant OR infants OR newborn* OR baby OR babies OR neonat* OR fetus OR pediatric* OR paediatric*)
CINAHL	(MH "Trypanosomiasis" OR cruzi OR chagas OR Trypanosom*) AND (MH "Infant, Newborn, Diseases+" OR MH "Disease Transmission, Vertical" OR OR prenatal OR gynecology OR pregnancy OR pregnant OR mother* OR congenital OR transplacental OR infant OR infants OR newborn* OR baby OR babies OR neonat* OR fetus OR pediatric* OR paediatric*)
EMBASE	('congenital chagas disease'/deOR (('chagas disease'/exp OR 'trypanosoma cruzi'/de OR 'trypanosomiasis'/de OR cruzi:ab,ti,kw OR chagas:ab,ti,kw OR trypanosom*:ab,ti,kw) AND ('congenital disorder'/lnk OR 'congenital infection'/de OR 'vertical transmission'/exp OR prenatal:ab,ti,kw OR gynecology:ab,ti,kw OR pregnancy:ab,ti,kw OR pregnant:ab,ti,kw OR mother*:ab,ti,kw OR congenital:ab,ti,kw OR transplacental:ab,ti,kw OR infant:ab,ti,kw OR infants:ab,ti,kw OR newborn*:ab,ti,kw OR baby:ab,ti,kw OR babies:ab,ti,kw OR neonat*:ab,ti,kw OR fetus:ab,ti,kw OR pediatric*:ab,ti,kw OR paediatric*:ab,ti,kw)))
IBSS	(cruzi OR chagas OR Trypanosom*) AND (prenatal OR gynecology OR pregnancy OR pregnant OR mother* OR congenital OR transplacental OR infant OR infants OR newborn* OR baby OR babies OR neonat* OR fetus OR pediatric* OR paediatric*)
JSTOR	(cruzi OR chagas OR Trypanosom*) AND (prenatal OR gynecology OR pregnancy OR pregnant OR mother* OR congenital OR transplacental OR infant OR infants OR newborn* OR baby OR babies OR neonat* OR fetus OR pediatric* OR paediatric*)
MEDES	(cruzi[título] OR cruzi[resumen] OR cruzi[palabras_clave]) AND (chagas[título] OR chagas[resumen] OR chagas[palabras_clave]) AND (tripanosom[título] OR tripanosom[resumen] OR tripanosom[palabras_clave])

Table S1. Databases and Equations used in Literature Search

Knowledge Production on Congenital Chagas Disease across Time, Borders and Disciplines: A Comprehensive Scoping Review
E.Rapp; M. Gold 2023

PUBMED	("Chagas Disease"[Mesh] OR "Trypanosoma cruzi"[Mesh] OR "Trypanosomiasis"[Mesh:NoExp] OR cruzi[tiab] OR chagas[tiab] OR Trypanosom*[tiab])<br>AND<br>("congenital"[Subheading] OR "Infant, Newborn, Diseases"[Mesh] OR "Infectious Disease Transmission, Vertical"[Mesh] OR prenatal[tiab] OR gynecology[tiab] OR pregnancy[tiab] OR pregnant[tiab] OR mother*[tiab] OR congenital[tiab] OR transplacental[tiab] OR infant[tiab] OR infants[tiab] OR newborn*[tiab] OR baby[tiab] OR babies[tiab] OR neonat*[tiab] OR fetus[tiab] OR pediatric*[tiab] OR paediatric*[tiab])
PSYCINFO	(cruzi OR chagas OR Trypanosom*) AND (prenatal OR gynecology OR pregnancy OR pregnant OR mother* OR congenital OR transplacental OR infant OR infants OR newborn* OR baby OR babies OR neonat* OR fetus OR pediatric* OR paediatric*)
SCIELO	(cruzi OR chagas OR Trypanosom*) AND (prenatal OR ginecología OR embarazo OR embarazada OR madre OR congénito OR transplacentaria OR bebé OR bébés OR recién nacido OR neonat* OR feto OR pediátric*)<br><br>(cruzi OR chagas OR Trypanosom*) AND (pré-natal OR ginecologia OR gravidez OR grávida OR mãe OR congénita OR transplacental OR criança OR crianças OR bebé OR bebés OR recém-nascido OR neonat* OR fetus OR pediátrica)<br><br>(cruzi OR chagas OR Trypanosom*) AND (prenatal OR gynecology OR pregnancy OR pregnant OR mother* OR congenital OR transplacental OR infant OR infants OR newborn* OR baby OR babies OR neonat* OR fetus OR pediatric* OR paediatric*)
SOCIOLOGICAL ABSTRACT	(cruzi OR chagas OR Trypanosom*) AND (prenatal OR gynecology OR pregnancy OR pregnant OR mother* OR congenital OR transplacental OR infant OR infants OR newborn* OR baby OR babies OR neonat* OR fetus OR pediatric* OR paediatric*)
TESEO	Search by title : (Chagas OR trypanosom OR cruzi)
WEB OF SCIENCE CORE COLLECTION	TS=("cruzi" OR "chagas" OR Trypanosom*)<br>AND<br>TS=("prenatal" OR "gynecology" OR "pregnancy" OR "pregnant" OR mother* OR "congenital" OR "transplacental" OR "infant" OR "infants" OR newborn* OR "baby" OR "babies" OR neonat* OR "fetus" OR pediatric* OR paediatric*)
GOOGLESCHOLAR	Keywords : (chagas OR cruzi OR trypano*) AND (congenital OR pregnan*)<br><br>Language selected: English, French, German, Portuguese and Spanish<br><br>Time selection (3) : No time limit; Since 2018; Since 2022. For each time selection the first 1000 sources were screened.

Note: In the Bibnet.org database, the Medline category has been removed.
